# Altered connection properties of the left dorsolateral superior frontal gyrus in *de novo* drug-naïve insomnia disorder

**DOI:** 10.3389/fnins.2025.1568557

**Published:** 2025-04-14

**Authors:** Hui Wang, Xianjun Ma, Xingru Xu, Qian Ning, Benyu Qiao, Bofeng Yang, Na Sun, Dong Xu, Xin Tang

**Affiliations:** ^1^Department of Neurology, Lianyungang Affiliated Hospital of Nanjing University of Chinese Medicine, Lianyungang, China; ^2^Department of Radiology, Lianyungang Affiliated Hospital of Nanjing University of Chinese Medicine, Lianyungang, China

**Keywords:** insomnia disorder, brain function, degree centrality, left dorsolateral superior frontal gyrus, drug-naïve

## Abstract

**Background:**

Insomnia disorder (ID) is increasingly prevalent, posing significant risks to patients’ physical and mental health. However, its neuropathological mechanisms remain unclear. Despite extensive research on ID using resting-state functional magnetic resonance imaging, a unified framework for describing its brain function alterations remains absent. Moreover, most prior studies have not fully accounted for the potential impact of medication on outcomes regarding enrollment criteria.

**Methods:**

We recruited 22 ID and 22 healthy controls (HC), matched for age and gender. Patients with ID were never prescribed medications for sleep disorders before enrollment. We detected differences in voxel-wise degree centrality (DC) between the two groups and analyzed the correlation between altered DC values and insomnia severity. Additionally, we conducted receiver operating characteristic analysis to evaluate the diagnostic effectiveness of the altered DC values for ID.

**Results:**

In ID patients, the weighted DC values of the left dorsolateral superior frontal gyrus (SFG) and the left supramarginal gyrus (SMG) were significantly lower than those of HC, with a notable negative correlation between the weighted DC values of the left dorsolateral SFG and PSQI scores. Receiver operating characteristic analysis showed that the weighted DC of the left dorsolateral SFG effectively differentiates between ID and HC, exhibiting high sensitivity and specificity.

**Conclusion:**

This study offers new insights into brain dysfunction and the pathophysiology of ID through voxel-based DC measurements. The results indicate that altered DC properties of the left dorsolateral SFG might serve as a diagnostic marker for ID and a potential therapeutic target for brain function modulation.

## 1 Introduction

Insomnia disorder (ID) is the most common sleep disorder and is linked to various pathological processes. Inadequate sleep duration and quality can negatively impact social, emotional, and cognitive functions, psychomotor performance, and metabolism, potentially leading to health issues like obesity, diabetes, cardiovascular diseases, and mental disorders (Fasiello et al., 2022). The global incidence of insomnia varies from 4 to 20% (De Crescenzo et al., 2022; Reimann et al., 2023; Morin et al., 2022), and it is on the rise year by year, which presents major economic and social challenges (Dopheide, 2020; Yan et al., 2018). Among the elderly population, this incidence even exceeds 50% (Wang et al., 2024; Proserpio et al., 2022). However, the precise neuropathological mechanisms behind this disorder remain poorly understood. Understanding the neuropathological changes in ID by examining brain function is crucial for investigating mechanisms of clinical efficacy and identifying potential therapeutic targets for these patients.

The rapid advancement of neuroimaging technologies has enabled the exploration of the neurobiological mechanisms underlying ID. Resting-state functional magnetic resonance imaging (rs-fMRI) has proven to be effective in identifying abnormal brain function in individuals with ID. Recent rs-fMRI studies have revealed functional alterations linked to the ID through measures such as low frequency amplitudelow frequency fluctuations (He et al., 2022; Li et al., 2016), regional homogeneity (Li et al., 2019), and the seed-based functional connectivity (FC) (Leerssen et al., 2019), finding that ID patients had lower ALFF in the left prefrontal cortex and left inferior parietal lobules, higher ReHo in the anterior cuneiform lobe, and stronger FC in the hippocampus and the left medial frontal gyrus. Despite a growing number of studies investigating anomalies in brain function related to ID, our understanding of its pathophysiology remains incomplete. Considering that the brain exhibits complex functional integration and information transmission networks (Wang et al., 2023), ID should be viewed as a global disorder rather than a localized one. Therefore, paying attention to functional networks is crucial for fully understanding the neuromechanisms involved in ID.

From the perspective of functional integration, the data-driven voxel-based degree centrality (DC) analysis avoids the subjective bias caused by the selection of regions of interest in the seed-based FC analysis, and offers a more objective method to detect abnormalities within the entire connectivity matrix of the whole brain functional connectome. Prior studies using this method identified abnormal intrinsic functional hubs in insomnia patients, showing elevated DC values in individuals within the right visual association cortex, right posterior cerebellum (Liu et al., 2018), and precuneus (Yan et al., 2018). A decreased DC was observed within the insula, left medial prefrontal cortex (Liu et al., 2018), left inferior frontal gyrus, and middle temporal gyrus (Yan et al., 2018). Only the decreased DC values showed significant correlations with clinical variables (Liu et al., 2018; Yan et al., 2018). However, these results were significant only if uncorrected for multiple comparisons. In general, studies on the characteristics of ID functional brain networks using DC as a data-driven method are limited, and no consistent conclusions have been reached thus far. Particularly, the current studies have not been able to account for the potential influence of therapeutic drugs on these findings. However, it should be noted that medical treatments can lead to reorganization of functional integration within brain networks and may confound interpretations regarding neuropathological mechanisms induced by diseases (Yan C. G. et al., 2019; Zhong et al., 2019). While the aforementioned studies required enrolled ID subjects to discontinue relevant therapeutic drugs for a period before participation to minimize treatment effects on results, the chronic effects of these drugs may still have caused or masked alterations in brain functional patterns in ID patients. Investigating drug-naïve ID patients could be more critical in elucidating the underlying mechanisms of the disease.

In the present study, we recruited de-novo unmedicated individuals with ID and applied a data-driven voxel-wise DC approach to analyze rs-fMRI data. We hypothesized that (1) early, untreated ID patients had alterations in the resting-state functional brain network, such as the destruction of the hub attribute in the left prefrontal lobe, (2) the abnormal DC values would be related to the severity of insomnia, and (3) a disrupted organization of the brain functional connectome characterizes ID, resulting in a decline in sleep. The altered DC features might contribute to understanding the neural basis of ID and exploring therapeutic targets.

## 2 Materials and methods

### 2.1 Subjects

The study was approved by the medical research ethics committee of Lianyungang Hospital of Traditional Chinese Medicine. Written informed consent according to the “Declaration of Helsinki” principles was obtained from all study participants. ID patients were recruited from the Neurology Department of Lianyungang Hospital of Traditional Chinese Medicine. The diagnosis was made by a senior neurologist according to the Diagnostic and Statistical Manual of Mental Disorders, Fifth Edition (DSM V). Meanwhile, a Pittsburgh sleep quality index (PSQI) score higher than 7 was required. The ID patients were also screened by an experienced psychiatrist according to the DSM V to exclude the diagnosis of depression, anxiety, and other psychiatric diseases at the same time. Patients were excluded if they had: (1) psychoactive medication use or psychiatric therapy prior to enrollment; (2) history of other psychiatric diseases; (3) history of cerebrovascular disorders, head injury, neurological surgery, or other neurologic diseases; (4) history of severe chronic diseases; (5) other sleep disorders (e.g., Breathing-Related Sleep disorder); (6) contraindications for MRI scanning; (7) incomplete clinical information. Healthy controls (HC), matched with the ID in age and gender, were recruited from the health examination center of the hospital and screened to exclude ID and psychiatric diseases. This study included 24 ID and 27 HC. However, seven subjects (nID = 2, nHC = 5) were excluded due to excessive head motion (a mean framewise displacement (FD) > 0.5 mm or head motions exceeding 3.0 mm translation or 3.0° rotation). This study eventually included 22 ID and 22 HC, which participated in the subsequent statistical analysis.

### 2.2 Clinical assessments

Demographic and clinical details were collected during the recruitment phase of the study. The insomnia severity was assessed using the Pittsburgh Sleep Quality Index (PSQI), which includes seven “component” scores: subjective sleep quality, sleep latency, sleep duration, habitual sleep efficiency, sleep disturbances, use of sleeping medication, and daytime dysfunction. The sum of these component scores yields a global score (Buysse et al., 1989), which is used as a score of insomnia severity for subsequent statistical analysis. All assessments and fMRI scans were performed on the same day as part of the study recruitment. The fMRI acquisition time was limited to 2:30–5:30 p.m. on the day of enrollment.

### 2.3 Acquisition of MRI data

MRI was performed on a 3 T MRI scanner (Discovery MR750, General Electric, Milwaukee, WI, USA). All participants were instructed to lay supine, remain as still as possible, close their eyes, and remain awake without thinking anything during the scan. Foam pads and a standard birdcage head coil were used to minimize head movement, and earplugs were used to attenuate the influence of noise. The parameters of 3D-T1 weighted imaging were as follows: repetition time = 8.2 ms; echo time = 3.2 ms; matrix = 256 × 256; fractional anisotropy = 12 degrees; field of view = 240 mm × 240 mm; slice thickness/gap = 1/0 mm; 136 slices covered the whole brain. Functional images were subsequently collected in the same slice orientation with a gradient-recalled echo-planar imaging pulse sequence, which included 240 volumes. The parameters were: repetition time = 2000 ms; echo time = 30 ms; flip angle = 90 degrees; matrix = 64 × 64; field of view = 240 mm × 240 mm; thickness/gap = 4.0/0 mm. For each subject, every rs-fMRI session lasted 480 s.

### 2.4 Preprocessing of rs-fMRI

All fMRI data were preprocessed with the Data Processing & Analysis toolbox for (Resting-State) Brain Imaging (DPABI Version 4.3^1^) (Yan et al., 2016), based on the MATLAB 2013b platform^2^. Specific steps can be found in the previous research (Wang et al., 2023).

The first ten volumes of each rest functional section were excluded for signal equilibrium and to allow participants to adapt to the scanning environment. Subsequently, the remaining images were corrected for slice timing using the middle slice as a reference and then realigned to remove head motion. The Diffeomorphic Anatomical Registration Through Exponentiated Lie Algebra algorithm was used to segment the T1 images into gray matter, white matter, and CSF (Ashburner and Friston, 2009). Several nuisance covariates, including the Friston-24 head motion parameters (regress out six head motion parameters, six head motion parameters from the previous time point, and the 12 corresponding squared items) (Friston et al., 1996), linear and quadratic trends, CSF and white matter signals, and were regressed out to minimize the motion artefact and the improve the signal-noise ratio. The resultant images were then normalized into the standard space by the Diffeomorphic Anatomical Registration Through Exponentiated Lie Algebra, resampled to 3 × 3 × 3 mm3 voxel size, and temporal band-pass filtered (0.01–0.08 Hz). To minimize the potential effects of head motion, we excluded participants with a mean FD > 0.5 mm or head motions exceeding 3.0 mm translation or 3.0° rotation (Navalpotro-Gomez et al., 2020). Finally, two ID and five HC were excluded. We then repeated the above preprocessing steps on fMRI data from 22 ID and 22 HC.

### 2.5 Degree centrality measurement

As a voxel-wise measurement for the whole-brain functional connectivity, degree centrality (DC) could reflect the global functional connections of brain hubs. The binary DC is defined as the number of edges connecting to a node. While the weighted DC refers to the sum of weights from edges connecting to a node (the node strength) (Zuo et al., 2012). The preprocessed fMRI data were used to calculate DC using DPABI Version 4.3. For each voxel, the time course was extracted and correlated with every other voxel in the brain. The whole brain connection matrix of each subject was constructed. In light of previous studies (Yan et al., 2018; Wang et al., 2020) which calculated DC separately for diverse correlation thresholds, the more commonly accepted correlation threshold r > 0.25 was adopted for binarization. Subsequently, the connection number was calculated to generate voxel-by-voxel DC, thereby eliminating potential spurious correlations that might stem from noises (Buckner et al., 2009). The matrix was transformed into a z-score matrix using Fisher’s r-to-z transformation to improve the normality. Finally, the resulting DC maps were spatially smoothed with a Gaussian kernel (full-width half maximum = 6 mm) (Li et al., 2017; Wang et al., 2020). The weighted version of DC was also computed.

### 2.6 Statistical analysis

All statistical analyses of the demographic, clinical variables, and mean FD were performed using the Statistical Product and Service Solutions version 19.0 (SPSS 19.0). After checking for normal distribution and homogeneity of variance within the data, the two-sample t-test was performed on the continuous data, and the chi-square test was used for the analysis of categorical variables. A *p*-value <0.05 was considered statistically significant.

The rs-fMRI data differences between ID and HC were evaluated in the DPABI statistical analysis module. The two-sample t-test within gray matter mask was performed to compare the differences in DC between the two groups. All statistical analyses were corrected according to the Gaussian random field (GRF) theory (voxel *p*-value <0.005, cluster *p*-value <0.05) using mean FD as covariates.

To investigate the relationship between neuroimaging abnormalities and the severity of insomnia in the ID group, The DC values of the significantly altered clusters were extracted for a Pearson correlation analysis with PSQI scores, which were further analyzed to examine their correlation with the component scores of the PSQI, Bonferroni corrected.

Receiver operating characteristic (ROC) curves were analyzed based on the DC values of the significantly altered clusters between ID and HC, to assess their value in the diagnosis and differentiation of ID. First, each significantly altered DC value between ID and HC was used as independent variables to establish a binary logistic regression model, respectively. The stepwise backward selection was used to identify indicators that were independent predictors of ID. The exclusion significance level was set to 0.1. Then, we computed the areas under the ROC curve (AUC) of each predictor that survived the logistic regression analysis. The 95% confidence intervals were calculated. We calculated the highest Youden index and evaluated the sensitivity and specificity of each factor.

## 3 Results

### 3.1 Demographic characteristics

The statistical analysis included data from 44 subjects: 22 ID and 22 HC. There were no significant differences between the two groups in terms of age and gender. ID group had significantly higher PSQI scores than the HC group (Table 1).

**Table 1:** Demographic, clinical characteristics and FD of two groups.

Groups	ID (*n* = 22)	HC (*n* = 22)	*p* value
Age, year	56.36 ± 11.75	52.00 ± 14.80	0.285
Gender, male/female	4/18	6/16	0.472
PSQI	11.77 ± 2.22	1.95 ± 1.59	**0.000**
Mean FD Jenkinson	0.08 ± 0.05	0.07 ± 0.04	0.360

Data are presented as mean ± standard deviation for continuous variables or frequencies for categorical ones. *p* values were obtained using two sample T test. *p* < 0.05 was considered significant and marked in bold.

### 3.2 fMRI results

No significant differences in mean FD were found between ID and HC groups (Table 1), indicating that the head motion did not affect the fMRI findings.

**Figure 1:**
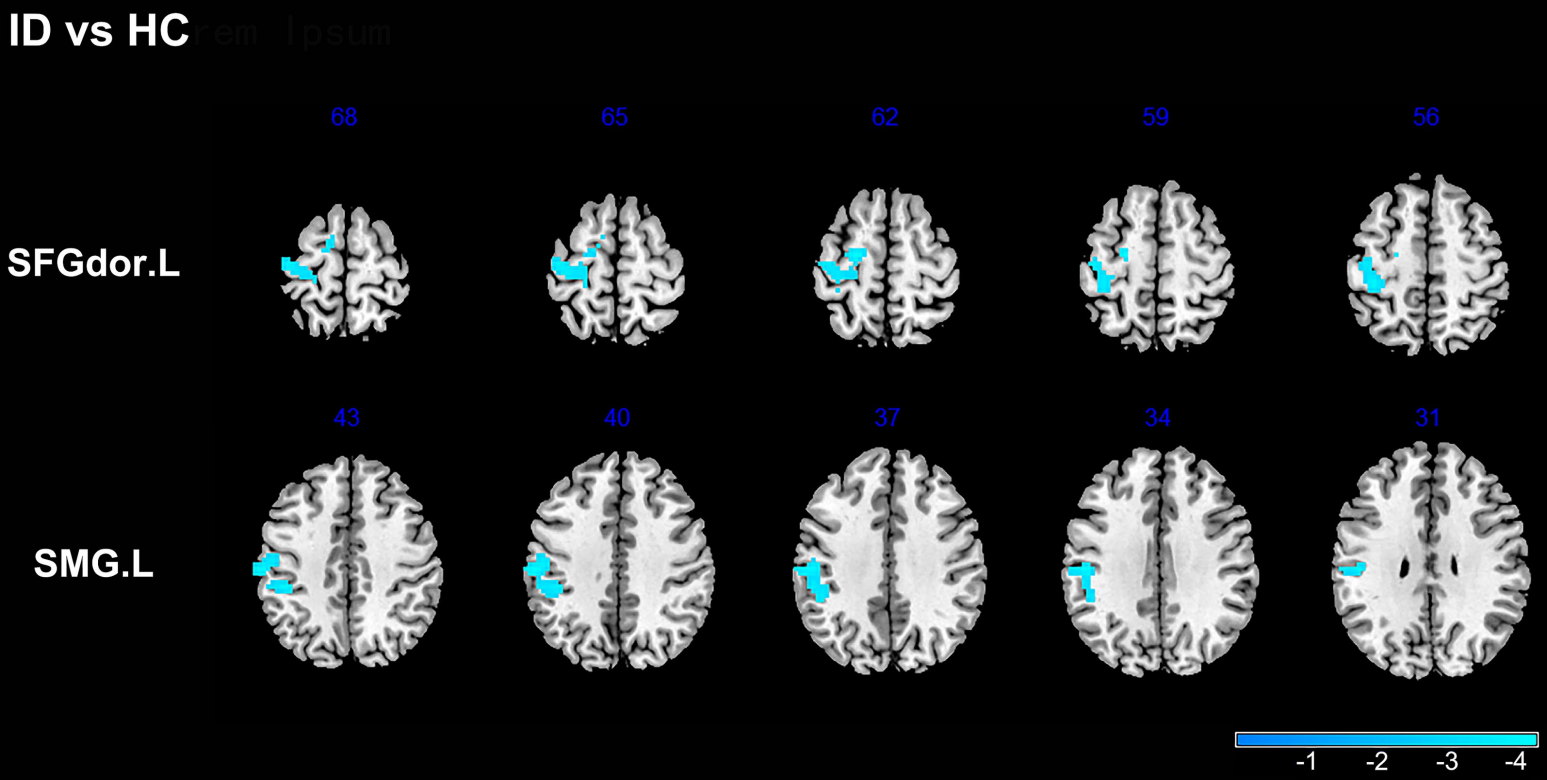
Group comparisons of the weighted DC maps between ID and HC groups. The color bar represents the *T* value of two-sample t-test with mean FD as covariates (GRF corrected, voxel *p*-value <0.005, cluster *p*-value <0.05). SFGdor.L, the left Superior frontal gyrus, dorsolateral; SMG.L, the left Supramarginal gyrus.

A significant group difference between the two groups in weighted DC was detected by a two-sample t-test in the left dorsolateral superior frontal gyrus (SFG) and the left Supramarginal gyrus (SMG). Compared to HC, ID showed a significant decrease in the weighted DC of the above regions (Table 2; Figure 1). However, there was no significant difference between the binary DC maps of the two groups.

**Table 2:** Difference in weighted DC of brain regions for patients between ID patients and HC.

Brain region (AAL)	L/R	BA	Clusters size (voxels)	Coordinates MNI			T Value
				*X*	*Y*	*Z*	
Superior frontal gyrus, dorsolateral	L	6	200	−21	−12	60	−4.2222
Supramarginal gyrus	L	3	180	−57	−21	39	−4.2693

All results were corrected by Gaussian random field (GRF) theory (voxel-level *p* < 0.005, cluster-level *p* < 0.05) with mean FD Jenkinson as covariates.

AAL, Anatomical Automatic Labeling; L/R, left/right; BA, Brodmann area; MNI, Montreal Neurological Institute.

### 3.3 Correlation analysis

**Figure 2:**
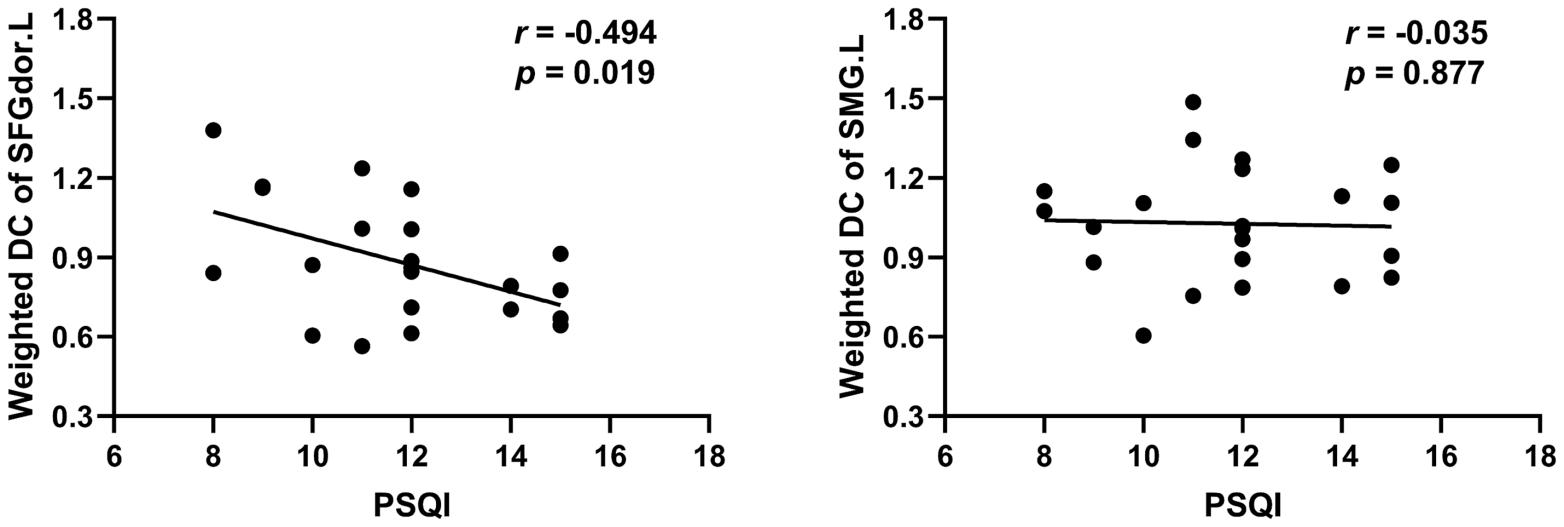
The correlation between the weighted DC values of the significant clusters and the PSQI scores. SFGdor.L, the left Superior frontal gyrus, dorsolateral; SMG.L, the left Supramarginal gyrus; PSQI, the Pittsburgh sleep quality index.

The weighted DC values of the left dorsolateral SFG showed a remarkably negative correlation with PSQI scores in the ID group (*r* = −0.494, *p* = 0.019). However, no significant correlation was recorded between the weighted DC values of the left SMG and the PSQI scores (Figure 2).

### 3.4 ROC analysis

**Figure 3:**
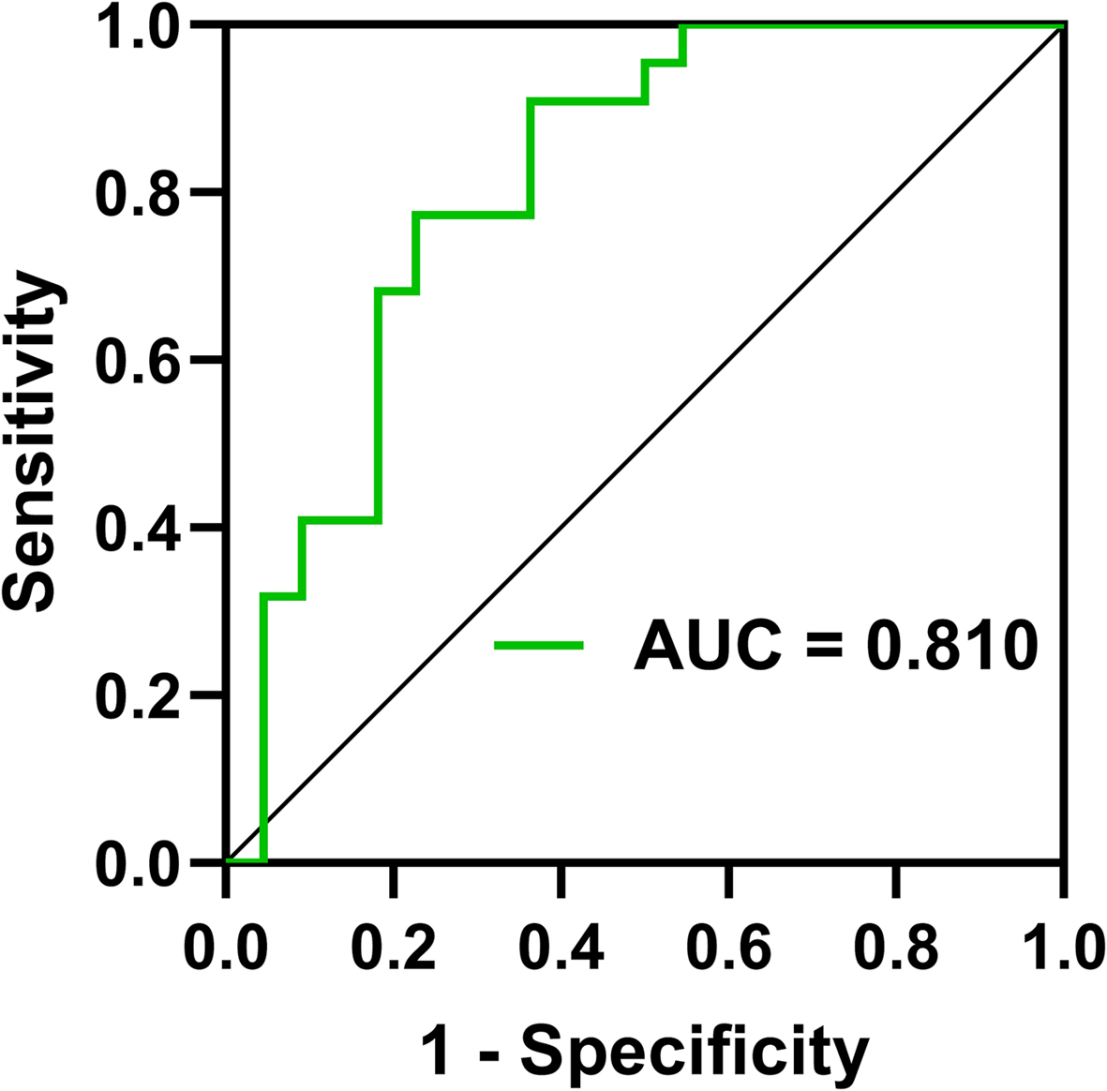
ROC curves for predicting ID by the weighted DC values of SFGdor.L. AUC, the areas under a ROC curve; ROC, receiver operating characteristic; SFGdor.L, the left Superior frontal gyrus.

The binary logistic regression models with ID as the dependent variables and the weighted DC values of the left dorsolateral SFG as the independent variable showed that the weighted DC value of the left dorsolateral SFG (*p* = 0.002) was an independent predictor of ID. Further ROC analysis was carried out to evaluate the diagnostic power of the predictor. The AUC value of the weighted DC values of the left dorsolateral SFG was 0.810(95%CI 0.678 ~ 0.942), *p* < 0.001 with sensitivity = 77.3%, specificity = 77.3% (Figure 3).

## 4 Discussion

The current study employed the data-driven DC analysis to investigate changes in functional brain networks associated with ID. Consistent with our hypothesis, significant differences in weighted DC were detected in the left dorsolateral SFG and the left SMG, with ID showing lower values in the above regions. In the ID group, the weighted DC of the left dorsolateral SFG was remarkably negatively associated with PSQI scores. Furthermore, the weighted DC value of the left dorsolateral SFG emerged as a potential marker in ID for its high sensitivity and specificity in distinguishing ID from HC.

The prefrontal cortex is more vulnerable to insomnia (Verweij et al., 2014). Chronic insomnia can impair the function of this brain region. He et al. (2022) found that patients with chronic insomnia showed reduced amplitude of low-frequency fluctuations and fractional amplitude of low-frequency fluctuations in the left dorsolateral prefrontal cortex compared to HC, suggesting that the initial state of this area may predict treatment efficacy. The dorsolateral prefrontal cortex has extensive connections with cortical and subcortical structures. Huang et al. (2017) observed FC strength reduction within the left basal ganglia/insula, right dorsolateral prefrontal cortex, right medial prefrontal cortex, and right cerebellum anterior lobe in ID patients, with the right medial prefrontal cortex negatively correlating with PSQI scores. Liu et al. (2018) found decreased FC between the left SFG and the left precuneus within the default mode network, suggesting that disconnection in the prefrontal cortex may significantly contribute to cognitive dysfunction in insomnia. It is worth mentioning that most of the previous studies using transcranial magnetic stimulation for insomnia selected the dorsolateral prefrontal cortex as the target site (Feng et al., 2019; Iseger et al., 2020). Overall, dysfunction of the prefrontal cortex is a key pathological feature of insomnia (Dai et al., 2016; Li et al., 2016; Zou et al., 2013), leading to impairments in the regulation of alertness, attention, and higher-order cognitive processes (Gong et al., 2020; Kober et al., 2008; MacDonald et al., 2000). This explains why people with insomnia are often sensitive to the environment and intrusive thoughts while trying to sleep, which is associated with an inability to fall asleep.

As a constituent of the prefrontal cortex, it is unsurprising that SFG had been wildly reported in previous fMRI studies of insomnia. Compared with female good sleepers, female chronic primary insomnia patients showed lower regional homogeneity of brain activity in the left SFG (Dai et al., 2016). Patients with primary insomnia showed decreased connectivity in regions of the right frontoparietal network, including the SFG (Li et al., 2018), and lower FC between the superior parietal lobe and SFG (Li et al., 2014). This investigation revealed a decreased DC value in the left dorsolateral SFG among individuals with ID, as well as an independent predictive effect of DC value of the left dorsolateral SFG on ID diagnosis. The left SFG receives projections from the visual, auditory, and somatosensory cortex, enabling the prefrontal cortex to integrate internal and external information to achieve top-down regulation of attention and affective states (Kaiser et al., 2015). We speculate that the decline in the functional attributes of the hub within the left dorsolateral SFG in ID might result in an excessive perception of negative information and the obstruction of top - down cognitive control (Kaiser et al., 2015; Ben Ezzdine et al., 2025), which further strengthens intrusive thoughts and uncontrolled concerns regarding insomnia, thereby promoting the maintenance and development of insomnia.

The SMG is located within the inferior parietal lobule, which is part of the DMN (Husbani et al., 2021) and plays an important role in the adjustment of consciousness (Marques et al., 2018). Previous studies have suggested that changes in DMN function are linked to hyperarousal symptoms in insomnia patients, often shown as increased DMN activity during the day and sleep stages. This heightened DMN activation before sleep is believed to enhance rumination and sleep-related worries, potentially obstructing the transition from wakefulness to sleep (Marques et al., 2015). The study revealed that the hub status of the left SMG in the brain functional network of ID patients was impaired, consistent with prior research. It is reported that decreased FC between the left SMG and the right amygdala in patients with insomnia was negatively correlated with the relative beta power of sleep EEG during stage N3, Kweon and colleagues speculate that the decreased FC reflect difficulty in cortical top-down regulation and cortical hyperarousal (Kweon et al., 2023). Structural MRI studies also showed a negative correlation between SMG white matter volume and PSQI scores (Bai et al., 2022), while greater SMG volume was linked to longer sleep duration (Cheng et al., 2021). Acupuncture treatment was investigated to enhance the functional connectivity of SMG.L in patients with ID (Chen et al., 2023; Zang et al., 2023), indicating that diminished functional connectivity of SMG.L may play a role in the pathological mechanism of ID. However, contrary results have also been reported. A recent study (Yin et al., 2024) indicated that increased FC between the right insular and the left SMG is positively correlated with poor sleep quality, anxiety, and depression in ID. Gong et al. observed increased locus coeruleus noradrenergic FC in the left SMG in the chronic insomnia disorder group (Gong et al., 2021). Variations in results may stem from the wide methodological heterogeneity, limitations in terms of study protocols, and different statistical approaches raised from the previous studies. In general, there is no unified finding and conclusion on the functional alterations of the left SMG in patients with ID. The results of this study did not show a significant correlation between the DC value of the left SMG and the severity of insomnia. As a key node of DMN, the role of left SMG in the neuropathological mechanism of insomnia remains to be further studied.

Several limitations deserve to be mentioned. First, this was a cross-sectional study, although it found that patients with ID had brain functional network features that differed from HC and speculated that these brain functional network changes played a role in the neural mechanisms of the disease. However, these alterations and their role in the pathogenesis of disease development need to be confirmed by more prospective follow-up studies. Second, the study had a comparatively limited sample size due to strict inclusion criteria aimed at eliminating the effects of drug treatment and other comorbidities. Additional data are required to validate and extend the findings for subsequent research. Third, this study adopted a slightly weaker voxel-level *p*-value threshold (*p* < 0.005). This represents a statistical limitation in the interpretation of the results. Given that we selected *de novo* drug - naïve patients with ID, the enrolled subjects might have a shorter disease duration or milder symptoms. The alterations in specific brain functional networks might be so slight that the differences between groups survived only under somewhat more lenient statistical thresholds. We would further enhance and validate these findings in future research endeavors. Fourth, the study lacked a quantitative assessment of depression and anxiety among the subjects. Anxiety and depression, often accompanied by insomnia, influenced the functional connectivity of DMN brain regions (Yan R. et al., 2019; Yu et al., 2021). While our subjects were screened by a psychiatrist to exclude those with anxiety and depression, patients with insomnia frequently experience these feelings. Consequently, fully eliminating these confounding effects is challenging. Future studies with quantification of anxiety and depression in individuals with ID are crucial for analyzing the relationship between the alterations in the brain functional network and the emotional scores. Additionally, they are essential for clarifying the role of emotional fluctuations within the neural mechanism of ID. At last, multi-channel sleep monitoring is essential for accurately evaluating objective sleep parameters. Future studies will adopt more precise methods to assess sleep differences between ID and HC.

## 5 Conclusion

Our study offers new insights into the dysfunction and pathophysiology of insomnia through voxel-wise DC measurement, enabling an unbiased exploration of abnormalities in the entire connectivity matrix of the full-brain functional connectome. Overall, evidence in this study supported the hypothesis that a disrupted brain functional connectome characterizes ID. The results identify functional deficits in two disrupted hubs, the left dorsolateral SFG, and the left SMG, with disturbed left dorsolateral SFG correlating with insomnia severity in the ID group. These findings underscore the importance of the left dorsolateral SFG in the neuropathological mechanisms of ID and enhance our understanding of ID’s functional characteristics. The DC signature of the left dorsolateral SFG holds promising potential as a diagnostic marker for ID. Moreover, it may function as a potential therapeutic target for interventions designed to modify brain function with the aim of alleviating insomnia. However, it must be emphasized that these findings are preliminary and require further research for confirmation and external data for verification.

## Data Availability

The data that support the findings of this article are available from the corresponding author via email upon reasonable request.

## Footnotes

^1^ http://rfmri.org/dpabi

^2^ http://www.mathworks.com/products/matlab
